# Hybrid Minimally Invasive Esophagectomy–Surgical Technique and Results

**DOI:** 10.3390/jcm8070978

**Published:** 2019-07-05

**Authors:** Jasmina Kuvendjiska, Goran Marjanovic, Torben Glatz, Birte Kulemann, Jens Hoeppner

**Affiliations:** Department of General and Visceral Surgery, Medical Center–University of Freiburg, Faculty of Medicine–University of Freiburg, 79106 Freiburg, Germany

**Keywords:** esophagectomy, minimally invasive surgery, hybrid procedures, esophageal cancer, surgical technique

## Abstract

Background: Hybrid minimally invasive esophagectomy (HMIE) has been proven to be superior when compared with open esophagectomy, with a significant reduction of postoperative morbidity. In HMIE, the laparotomy is replaced by a minimally invasive laparoscopic approach. The radical mediastinal resection plus reconstruction is performed by a thoracic approach through a muscle-sparing thoracotomy. In this instructional article, we describe the surgical technique of HMIE in detail in order to facilitate possible adoption of the procedure by other surgeons. In addition, we give the monocentric results of our own practice. Methods: Between 2013 and 2018, HMIE was performed in 157 patients. The morbidity and mortality data of the procedure is shown in a retrospective monocentric analysis. Results: Overall, 54% of patients had at least one perioperative complication. Anastomotic leak was evident in 1.9%, and a single patient had focal conduit necrosis of the gastric pull-up. Postoperative pulmonary morbidity was 31%. Pneumonia was found in 17%. The 90 day mortality was 2.5%. Wound infection rate was 3%, and delayed gastric emptying occurred in 17% of patients. In follow up, 12.7% presented with diaphragmatic herniation of the bowel, requiring laparoscopic hernia reduction and hiatal reconstruction and colopexy several months after surgery. Conclusion: HMIE is a highly reliable technique, not only for the resection part but especially in terms of safety in reconstruction and anastomosis. For esophageal surgeons with experience in minimally invasive anti-reflux procedures and obesity surgery, HMIE is easy and fast to learn and adopt.

## 1. Introduction

Hybrid minimally invasive esophagectomy (HMIE) has recently been proven to be superior when compared with open esophagectomy (OE). A significant reduction of postoperative morbidity has been shown in the largest multicentric randomized controlled trial (RCT) done on minimally invasive esophagectomy [1]. In HMIE, the laparotomy is replaced by a minimally invasive laparoscopic approach. The radical mediastinal resection plus reconstruction is performed by a thoracic approach through a muscle-sparing thoracotomy. European clinical cancer treatment guidelines have already included HMIE as “the standard procedure” for surgical resection of esophageal cancer [2]. In our own practice, HMIE was started in 2013 and very quickly became the standard procedure for the surgical treatment of non-metastatic esophageal cancer. We have experienced a very high safety and excellent results in our patients, with a steep learning curve associated with no extra learning-curve-associated treatment morbidity. In this instructional article, we give the results and describe the surgical technique of HMIE in detail in order to facilitate possible adoption of the procedure by other surgeons. Especially esophageal surgeons with experience in minimally invasive anti-reflux procedures and obesity surgery are familiar with the anatomic upper GI environment of the operative procedure. For them HMIE will be easy and fast to learn and adopt.

## 2. Patients and Methods

At the Medical Center of the University of Freiburg, HMIE is regularly applied for the surgical treatment of esophageal cancer since 2013. From October 2013 to April 2018, overall, 157 HMIE procedures have been carried out. In this article, we show the morbidity and mortality data of the procedure and describe the surgical technique in detail. All procedures performed were in accordance with the ethical standards of the institutional research committee and with the 1964 Helsinki declaration and its later amendments or comparable ethical standards. Informed consent was obtained from all individual patients included in the study. The study was approved by the Medical Ethics Committee of the University of Freiburg (File No. 253/19).

## 3. Pretherapeutic Work-Up

The decision for an HMIE procedure was made based on preoperative contrast-enhanced computed tomography (CT) imaging, comorbidities, and informed consent. Comorbidities were recorded, and pulmonary and cardiac check-up were routinely performed in risk patients. Besides the recommended breathing and physical exercises, no special structured prehabilitation program for a special group of patients was applied in the analyzed collective. Pretherapeutic diagnostics included endoscopy and thoraco-abdominal CT in all patients. Endoscopic ultrasound was done routinely for staging if technically possible in esophageal carcinoma.

## 4. Multimodal Treatment

All tumor patients were discussed in our multidisciplinary tumor board, and decision for neoadjuvant treatment was made when the T stage was T3 or T4 and/or lymph nodes were suspected to be positive and patients had no contraindications for multimodal treatment. Perioperative chemotherapy was performed according to the FLOT protocol for adenocarcinoma of the esophagus and esophagogastric junction [3]. Neoadjuvant chemotherapy protocols were scheduled for adjuvant continuance starting 4–8 weeks after the operation, with the same drug composition and dosage as preoperatively. Neoadjuvant chemoradiation was performed for squamous cell tumours and adenocarcinoma according to the CROSS protocol [4]. From 2013 to 2015, locally advanced esophageal adenocarcinomas (cT3/T4 and/or cN+, M0) with distal extent of tumor at or below the Z-line were treated with perioperative chemotherapy, and those with distal extent of tumor cephalad to the Z-line were treated with neoadjuvant chemoradiaton. From 2016 to 2018, the decision was made by randomization for either FLOT perioperative chemotherapy or CROSS neoadjuvant chemoradiaton in the ESOPEC trial (NCT02509286). After neoadjuvant chemoradiation or neoadjuvant chemotherapy, the tumor patients were restaged by endoscopy and computerized tomography, and resection was performed approximately 6 weeks after the end of neoadjuvant treatment.

## 5. Surgical Technique

### 5.1. Technical Setup

**Figure 1:**
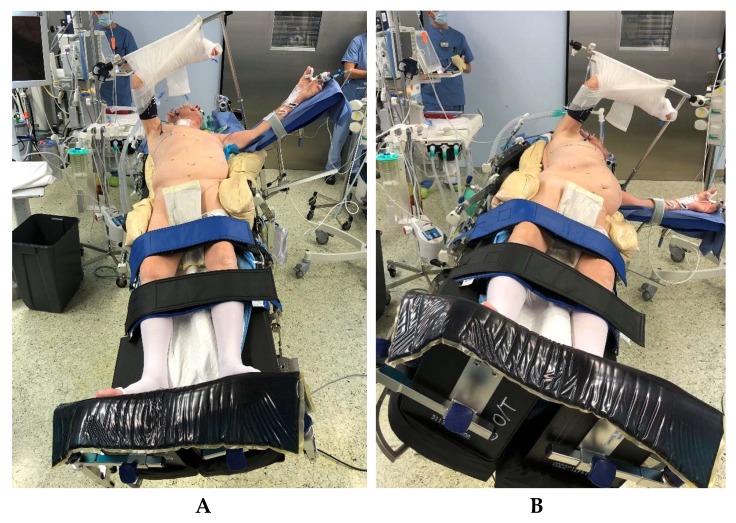
Positioning of the patient on the operating table. **A**. Position for laparoscopic part of hybrid minimally invasive esophagectomy (HMIE). **B**. Position for thoracic open part of HMIE.

In addition to double-lumen-tube ventilation, a central venous catheter, an arterial access for blood gas controls and continuous blood pressure monitoring, and a thoracic peridural catheter for intra- and postoperative analgesia are routinely used. For patient positioning (Figure 1), an operating table/column system is used which enables lateral tilting of 25° in both directions as well as anti-Trendelenburg positioning of up to 40°. A vacuum mat, 100 cm long, with lateral recesses at the level of the thoracotomy is also used. For the laparoscopic dissection, a Ligasure^®^ (Medtronic GmbH, Meerbusch, Germany) device 5 mm–37 cm with Dolphin or Maryland Tip is used during the procedure.

### 5.2. Positioning of the Patient

The patient is positioned on a vacuum mat with additional side supports at both iliac crests and on the right shoulder, in a slightly twisted position (Figure 1).

In this, the right chest side is elevated with the pelvis flat. In order to enable a safe anti-Trendelenburg positioning for the laparoscopic part of the operation, leg straps are placed on the upper and lower calf and fixed foot-boards are used. This positioning makes it possible to perform the laparoscopic part of the operation with the table initially tilted legs down and toward the right, providing very good exposition of the upper abdomen (Figure 1a). Subsequently, the operating table needs only to be tilted toward the left and horizontally and a lateral thoracotomy can be performed with good exposition of the right-sided mediastinum for the esophagectomy and reconstruction after gastric pull-up, without time-consuming repositioning of the patient (Figure 1b).

## 6. Laparoscopic Abdominal Operation Step

### 6.1. Trocar Placement

**Figure 2:**
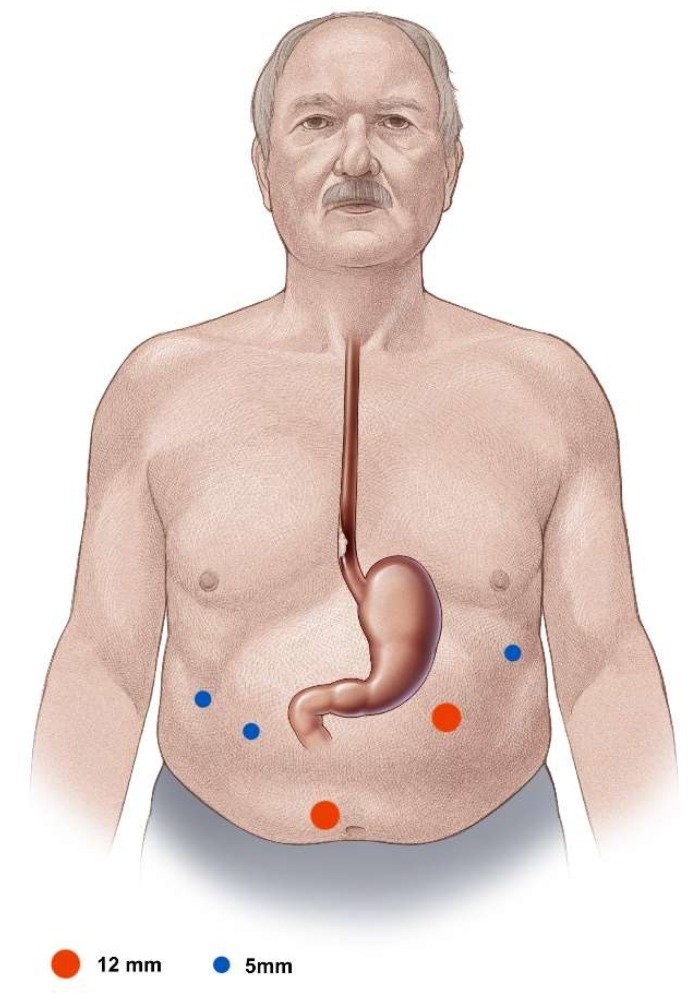
Trocar placement.

For the laparoscopic segment of the operation, the surgeon stands at the patient’s right side and the assistant operating the camera opposite, on the left. In addition to the paraumbillical camera trocar, one 12 mm working trocar, one 5 mm working trocar, and two additional 5 mm holding trocars are placed (Figure 2). In obese patients, the camera trocar is placed clearly cephalad to the umbilicus in the midline with the working trocars placed also proportionally cephalad. The capnoperitoneum is set up with a pressure of 13–15 mmHg.

### 6.2. Exploration and Exposition

After exploration of the abdominal cavity and visual exclusion of peritoneal or superficial hepatic metastatic spread, the left hepatic lobe is retracted in ventrocranial direction using a hepatic retractor, inserted through the right eccentric 5 mm trocar.

### 6.3. Crural Dissection

After division of the gastrohepatic ligament at the left edge of the hepatoduodenal ligament, the right pillar of the crus is exposed and the distal esophagus separated from it. After division of the phrenoesophageal membrane at the anterior hiatal commissure, the left pillar of the crus is divided from the esophagus. Now the abdominal esophagus is mobilized circularly, and mobilization of the upper gastric fundus at the angle of His is carried out by dividing the peritoneal attachment.

### 6.4. Abdominal and Lower Mediastinal Lymphadenectomy

**Figure 3:**
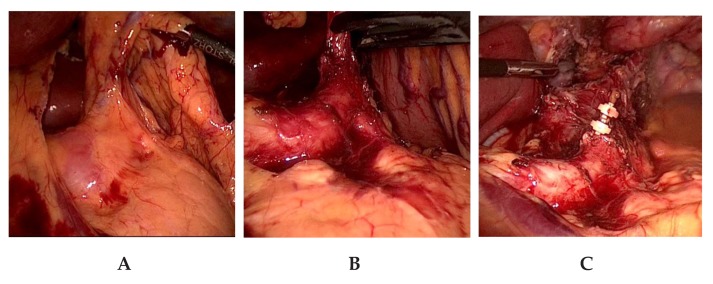
Abdominal and lower mediastinal lymphadenectomy. **A**. Anterior exposure. The axis of the left gastric artery is orientated upwards by the assistant. **B**. Lymphadenectomy at hepatic artery and celiac trunk. **C**. Situs after ligation of the left gastric artery, incision of the right pillar of the crus, lower mediastinal lymphadenectomy, and entry into the right pleural cavity.

The abdominal lymphadenectomy is carried out from an anterior approach. AJCC classified lymph node station numbers 8Lo, 9R, 15, 16, 17, 18, 19, and 20 are resected in the abdomen and the lower mediastinum from the laparoscopic abdominal and transhiatal approach [5]. To facilitate the exposure of the lymph nodes located at the branches of the celiac trunk and the pancreatic upper border, the axis of the left gastric artery is retracted upwards in the direction of the ventral abdominal wall (Figure 3a,b). In visceral obesity, the dissection of the fatty tissue at the lesser curvature is done prior to lymphadenectomy of lymph node station numbers 18, 19, and 20. Lymphadenectomy is facilitated by this sequence. After lymphadenectomy at the hepatic artery, the celiac trunk, the splenic artery, and the left gastric vessels, the latter are ligated and transected near their origin with PDS-clips (Figure 3c). This completes the lymphadenectomy at the celiac trunk. We try to preserve any broad-caliber replaced left hepatic artery arising from the left gastric artery, which is present in ca. 10% of the cases. In these cases, only the gastric branches of the left gastric artery are ligated with PDS-clips and the trunk of the left gastric artery along with the replaced left hepatic artery are preserved. Nonetheless, a thorough lymphadenectomy of the lymph nodes is performed on the left gastric artery. In our experience, this is laparoscopically feasible without problems and requires only slightly more time. The esophageal hiatus is then expanded by a incision of the right pillar of the crus, followed by esophageal mobilization and an en-bloc lymphadenectomy in the lower mediastinum to about 5–7 cm above the level of the diaphragm (Figure 3c). The incision of the right crus facilitates radical lower mediastinal lymphadenectomy and allows a straight descending course of the gastric tube. In large hiatal hernia, this step is not performed as the size and formation of the hiatus does not handicap lower mediastinal lymphadenectomy or straight descent of the gastric tube.

### 6.5. Laparoscopic Mobilization of the Stomach

For gastric mobilization, the gastrocolic ligament is split under careful sparing of the gastroepiploic arcade beginning at the level of the gastric corpus. In order to achieve adequate mobilization of the distal stomach, a short mobilization of the gastric antrum on the large curvature side is usually performed from left to right. From that point, the gastrocolic ligament is divided from right to left in the direction of the lower pole of the spleen. The left gastroepiploic vessels are ligated close to their origin at the branches with the splenic vessels. To improve venous drainage of the later gastric conduit, care is taken that an omental flap at the level of the splenic flexure remains with the gastroepiploic arcade. This preserves the venous collaterals between the right and left gastroepiploic arcades. In progressive dissection of the gastrolienal ligament, the dissection level above the splenic hilum reaches increasing proximity to the gastric wall in the area of the gastric fundus. After complete mobilization of the greater curvature, the gastric fundus is held in the direction of the patient’s right shoulder and the remaining retrogastric embryonal adhesion strands are divided. After gastric mobilization, the pylorus can be raised without strain to the level of the right pillar of the crus.

### 6.6. Endoscopic Pylorus Dilatation

After the stomach is completely mobilized, intraoperative endoscopic pyloric balloon dilatation is performed for 2 min using a 20 mm balloon to prevent postoperative pylorospasm, which frequently becomes clinically manifest after vagotomy within the context of esophagectomy.

### 6.7. Closure of the Abdomen

After subsequent control of the anatomically correct position of the mobilized stomach for later gastric pull-up, a drain is positioned through the left 5 mm trocar incision to the upper edge of the pancreas and splenic hilum. The remaining trocars are removed under visual control, and the trocar insertion sites are closed.

## 7. Open Thoracic Operation Step

### 7.1. Muscle Sparing Right Lateral Thoracotomy

**Figure 4:**
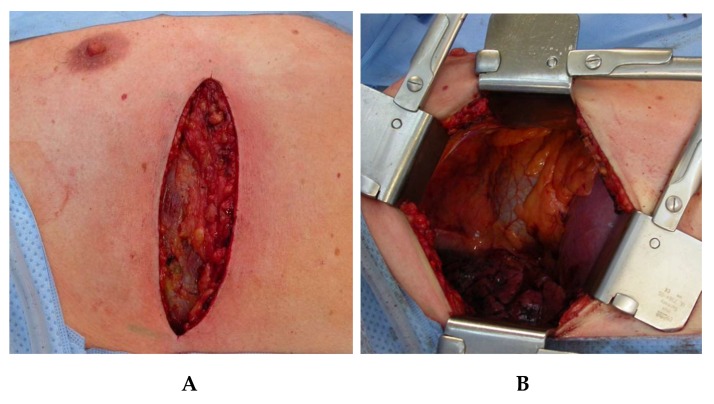
Muscle-sparing right lateral thoracotomy at fifth intercostal space. **A**. Positioning and extend of the incision. **B**. Exposition after introduction of the surgical rip retractor.

After finalization of the laparoscopic part of the operation, the patient is repositioned by adjusting the operating table to a horizontal position, tilted at 25° to the left (Figure 1b). Access to the right pleural cavity and to the mediastinum is created by means of a muscle-sparing lateral thoracotomy along the fifth right intercostal space (Figure 4). Both latissimus and serratus muscle are preserved without division. After deflation and removal of the right lung from ventilation, the right-sided inferior pulmonal ligament is divided and the right lung is exposed toward ventral, enabling clear access to the whole length of the thoracic esophagus.

### 7.2. En-Bloc Esophagectomy

After incision of the mediastinal pleura at both sides of the azygos arch, this is ligated and divided near its entry into the superior vena cava. Starting at the level of the former azygos arch, the esophagus is now dissected en-bloc with the attached lymph and fatty tissue down to the diaphragm. From the thoracic incision, lymph node station numbers 8M and 7 are resected in the mediastinum in tumors located in the distal esophagus. Lymph node station numbers 8U, 4R, and 4L are added if the tumor extends to the middle or upper thoracic esophagus [5]. The ascending azygos vein is preserved, and the adventitia of the descending aorta and the left pleura mediastinalis limit the plane of preparation. The thoracic duct is held for en-bloc operation specimen and ligated above the level of the diaphragm. This enables reliable avoidance of a postoperative chylothorax. The level of the lower mediastinal esophagus, already dissected during laparoscopy is reached. The esophagus is already circularly freed in this region. The carinal lymph nodes are held en-bloc to the operative specimen and are completely removed. For adenocarcinoma, the esophageal transsection is usually performed 2 cm above the former azygos arch. For squamous cell carcinoma, esophageal resection is carried out up to the superior thoracic aperture. The esophagus is transsected after a purse-string suture has been rendered by a purse-string clamp (Ethicon EH40) at the oral esophageal stump.

### 7.3. Gastric Pull-Up and Construction of the Gastric Conduit

Through the expanded esophageal hiatus, the laparoscopically mobilized stomach is pulled up into the right pleural cavity with respect to appropriate rotation and without compromising or injuring the supplying gastroepiploic arcade. Starting at the gastric fundus approximately 3 cm distant from the angle of His, the gastric cardia with the attached esophagus is removed using several cartridges of a flexible 60 mm linear stapling device (Endo-GIA, 3.8 mm staples), thus forming a gastric conduit about 4 cm in diameter. The small curvature of the stomach, with attached lymph and fatty tissue is largely resected, and the right gastric artery with its antral branches are preserved. To prevent later bleeding and gastric wall hematomas, the linear staple row at the small curvature side is oversewn with a running suture (PDS 4/0). This results in a tubular gastric conduit reaching at least into the superior thoracic aperture. The operative specimen, which consists of the en-bloc resected esophagus with the gastric cardia and the attached abdominal and mediastinal lymph nodes is given for intraoperative frozen section analysis.

### 7.4. Intrathoracic End-to-Side Esophagogastrostomy

**Figure 5:**
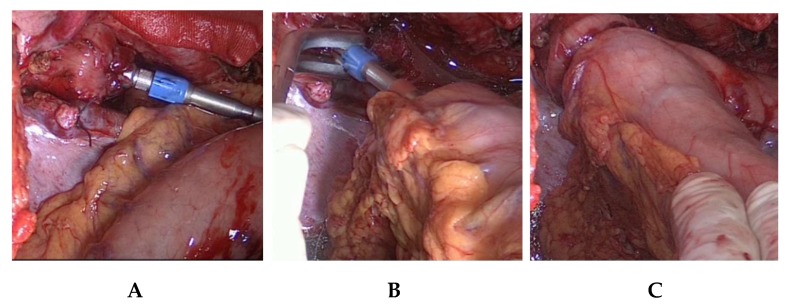
Procedure of circular stapled end-to-side esophagogastrostomy (**A**–**C**).

To perform the end-to-side esophagogastrostomy, the anvil of a 25 mm or 28 mm circular stapler, dependent on the diameter of the oral esophageal stump is inserted in the latter (Figure 5).

**Figure 6:**
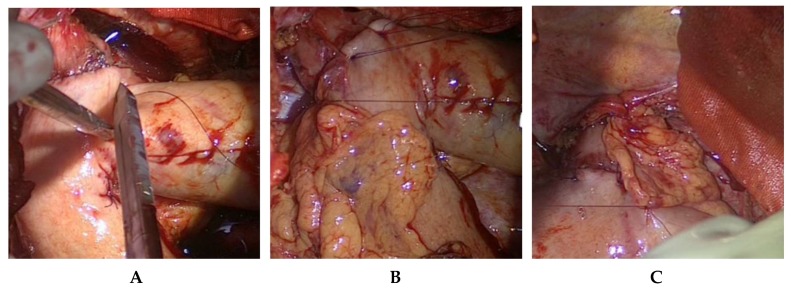
Oversewing of circular stapled esophagogastrostomy (**A**,**B**) and semifinished anastomotic omentopexy (**C**).

The localization of the esophagogastrostomy on the large curvature is determined at the tubular gastric conduit. The optimal anastomotic location guarantees a straightly stretched but still tension-free course of the gastric conduit. Since the gastric conduit usually extends considerably beyond the selected level, the gastroepiploic arcade is removed above and in the area of the future anastomosis near the gastric wall, whereby no problems need be anticipated in arterial or venous perfusion (Figure 5). The circular stapler device is inserted through a gastrotomy created at the overlapping tip of the gastric conduit and the end-to-side esophagogastrostomy carried out on the large curvature side after the stapler device is connected. Additional anastomotic stability is achieved by interrupted circular all-layer oversewing (PDS 4/0) of the anastomosis and circular anastomotic wrapping with the adjacent greater omentum (Figure 6).

The tip of the gastric conduit extending cranial to the esophagogastrostomy is removed with a flexible 60 mm linear device (3.8 mm staples), and the resultant linear staple row is also oversewn (PDS 4/0). Finally, a nasogastric tube is inserted to the level of the diaphragm.

### 7.5. Placement of Chest Tubes and Thoracic Wound Closure

After final control and irrigation of the thoracic situs, two chest tubes are placed at the right chest and the right lung is re-inflated after rib re-adaption sutures have been made. To prevent postoperative atelectasis, attention is paid to complete expansion of the lung. If necessary, the position of the pulmonary lobes can be corrected manually prior to closing the thoracotomy, and thus provide for complete ventilation of all segments. After continuous re-adaptation sutures of the intercostal and serratus musculature have been made, finally the skin is sutured. Since left-sided pleura effusions often occur postoperative–even though the left-sided pleura is not opened in mediastinal dissection–finally, a left chest tube is placed under digital control through a 2 cm mini thoracotomy.

## 8. Postoperative Management

After extubation of the patient in the operating room, a 4 to 5 day period follows for postoperative cardiorespiratory monitoring and for guarantee of optimal postoperative medical pain management on the intermediate care unit (IMC). The analgesic treatment is done via the inserted peridural catheter, which is usually removed after 5 days. Overlapping, the analgesic therapy is switched to intravenous and oral medication. Breathing exercises are started on the first postoperative day. Also on the first postoperative day, patients begin to drink while the nasogastric tube is still in place. On the 5th postoperative day, a contrast swallow is routinely used to rule out delayed gastric emptying. If the gastric conduit is drained adequately, the nasogastric tube is removed and the oral diet is begun with soft and smooth food. In cases of persistent gastric emptying disorder, endoscopic pyloric dilatation is performed on the 10th postoperative day, and the removal of the nasogastric tube is scheduled as a function of its drainage balance. The underlying chest drains are also removed depending on their drainage balance and in the absence of air fistula between the 2nd and 7th postoperative days. Patients may be hospitalized between the 7th and 14th postoperative days, and the first outpatient visit, including routine esophagogastroscopy, is scheduled for 6 weeks postoperatively. If an anastomotic stenosis becomes noticeable in this examination, endoscopic anastomosis dilatation is done. In the case of reflux-related esophagitis, which is diagnosed in about 10% of patients, proton pump inhibitor therapy is initiated, often permanently.

## 9. Results

Between October 2013 and April 2018, we performed 157 HMIE. Two different surgeons carried out the HMIE procedures. Overall, 155 procedures have been performed for esophageal tumors and two were done in complicated gastroesophageal reflux disease. Locally advanced (cT3-4cNx; cT2N+) tumor disease was pretherapeutically diagnosed in 122 (78%) of the patients. Total minimal-invasive esophagectomy (TMIE) and OE were done in 61 patients in the reporting period. The demographic data of the HMIE collective is shown in Table 1.

The median operating time was 290 minutes. In two patients, conversion to laparotomy was necessary due to severe intraabdominal adhesions. Medium intermediate care and hospital stay was five and 14 days. Patients were regularly extubated in the operating room and directly sent to the surgical IMC. No regular intensive care unit (ICU) therapy or postoperative respirator therapy was scheduled. In three patients, direct postoperative respirator therapy with delayed extubation on the ICU was necessary. Another nine patients were admitted from the IMC to the ICU due to postoperative respiratory insufficiency. We had six re-admissions to the ICU after transfer to the surgical ward. Overall, 18 patients were readmitted to the hospital after discharge from hospital. The procedural and hospital stay parameters are given in Table 2.

Overall, 54% of patients had at least one perioperative complication. The 90 day mortality was 2.5%. The three casualties were caused by chemotherapy-induced pulmonary fibrosis with pneumonia and postoperative respiratory insufficiency in one patient, postoperative aortic arrosion with fatal haemorrhagic shock three weeks after surgery in another patient, and one septic death caused by an anastomotic leak with fail to rescue. Surgical complications were diagnosed in 29% of patients. Three patients had an anastomotic leak requiring endoscopic vacuum treatment, and another single patient had focal conduit necrosis of the gastric pull-up, managed by endoscopic vacuum treatment. For anastomotic leak and conduit necrosis, the Esophageal Complications Consensus Group (ECCG) consensus definitions were applied [6]. Anastomotic leak was defined as full thickness gastrointestinal defect involving esophagus, anastomosis, staple line, or conduit irrespective of presentation or method of identification. In the present series, we had three Type 2 leaks diagnosed at postoperative day 5, 7, and 9 by CT in all three cases. All three were verified by endoscopy. One Type 1 gastric conduit necrosis was diagnosed at postoperative day 9 by endoscopy. The first patient with an anastomotic leak was done in 2017, as the 106th patient of this series. The other two leaks occurred also in 2017, in patient No. 111 and 130. In four patients, mucosal ischemia of the tip of the gastric conduit was found and managed conservatively. Wound infection rate was 3%, and delayed gastric emptying occurred in 17% of patients. Delayed gastric conduit emptying was diagnosed in patients requiring postoperative endoscopic pyloric dilatation, in patients with delayed discharge due to symptoms of delayed gastric emptying, and in patients requiring prolonged nasogastric drainage more than one week postoperative. Pulmonary infections occurred in 17% of patients. We had 44 patients with overall 56 complications requiring surgical or interventional therapy (Clavien/Dindograde 3a/b). These were: two mucosal ischemia, three anastomotic leaks, one gastric tube necrosis, 27 delayed gastric conduit emptying, three wound infections, five chylothorax, 12 pleural effusion, three cardial complications requiring intervention. Additional to the perioperative complications, 20 patients (12.7%) presented with enterothorax and herniation of the transverse colon, requiring laparoscopic re-exploration, hernia reduction, and colopexia. Five patients presented as a surgical emergency. All 20 were discovered by CT and presented 7 days to 37 month postoperatively.

The surgical outcome and the postoperative complications are displayed in Table 3.

## 10. Discussion

The advent of minimally invasive surgery in esophageal cancer treatment has led to a wide distribution of different minimally invasive techniques of esophagectomy. We report on the technique of HMIE and give results of routine utilization in the environment of a specialized centre with high-volume treatment. In the past, HMIE has already been described as a technique with a potentially comparable benefit for the patient regarding postoperative pulmonary morbidity [7,8,9]. In our own practice, we found postoperative pulmonary morbidity in 31% of the patients in the series reported here. Concerning the most severe reconstructive complications, only 2% suffered from anastomotic leakage and only one single patient experienced gastric conduit necrosis. Moreover, we recognized HMIE as a technique that is relatively easy to adopt for surgeons with experience in OE and laparoscopic anti-reflux surgery. We did not notice any relevant learning-curve-associated extra mortality in HMIE. The results of a multicentric randomized controlled trial (RCT) comparing hybrid minimally invasive laparoscopic thoracotomic esophagectomy (HMIE) with OE have been published recently [1]. Another smaller multicentric RCT comparing TMIE with OE was published in 2012 [10]. Both trials were able to show significant benefits concerning short-term outcomes for the minimal-invasive approaches. Another monocentric trial compared robot-assisted TMIE with OE [11]. Especially pulmonary complications decreased by the utilization of minimal-invasive approaches TMIE and HMIE. No prospective randomized controlled trials are available comparing one minimal-invasive technique with the other. TMIE is a promising procedure, but the technically demanding reconstruction, long operating time, and a lack of thoracoscopic operation experience of many gastrointestinal surgeons make the procedure difficult and longsome to adopt [12]. Moreover, long and stretched learning curves have to be expected. Main criticism of TMIE is that the advantages of the minimally invasive approach with especially reduction of pulmonary complications are bought dearly with higher rates of severe reconstructive complications like anastomotic leaks and gastric conduit necrosis [13].

Recently, a multicentre propensity score analysis based on the Dutch national esophagectomy registry compared 433 TMIE versus 433 OE. In presence of a postoperative pulmonary morbidity rate of 35% for TMIE, the study showed significantly increased rates for anastomotic leak (21% vs. 15%), gastric conduit necrosis (3% vs. 0%), and re-intervention rate (28% vs. 21%) for TMIE. Especially in the subgroup of Ivor Lewis esophagectomy with intrathoracic anastomoses, the anastomotic leak rate was increased for thoracoscopic esophagosgastrostomy compared with that of esophagosgastrostomy carried out via the thoracotomy approach (21% vs. 10%; *p* = 0.001) [9]. Another multicenter European study analyzed the outcomes of TMIE carried out in six specialized centers with a treatment volume of >20 esophagectomies per year. In this study, an anastomotic leak of 15% was reported for intrathoracic anastomoses [14]. In both studies, 30 days mortality rates of 4% and 2% were reported for TMIE [13,14]. These results indicate up-to-date unsolved safety issues in the reconstructive part of TMIE. Finally, another multicentre retrospective study from four European specialized centres (>40 TMIE/year) analysing 646 Ivor-Lewis TMIE, was able to describe a “plateau” of 8% anastomotic leakage rate after passing the learning curve of TMIE. The length of the learning curve to reach this result was calculated as 119 cases per hospital in this analysis [15]. The here reported results are limited by the retrospective and monocentric analysis, although we report on the so far largest published series of HMIE with 157 patients treated over a period of 55 month. In comparison, a series of 103 patients treated over a period of 31 months in the prospective multicentre French MIRO trial reported an anastomotic leak rate of 11%, a rate of conduit necrosis of 2%, and a 30 days mortality rate of 1% [1]. The reported rate of postoperative enterothorax in our patients was 12.7% in the analyzed period. The finding corresponds with the rates given in other literature. In a large European series of open and minimally invasive esophagectomy in 45 of 488 (9%) patients (who underwent systematic postoperative CT scans, like our patients), enterothorax was evident [16].

Besides, the different techniques of minimally invasive esophagectomy especially enhanced recovery after surgery (ERAS) protocols, incorporating one or more elements of preoperative prehabilitation, pain relief, early mobilization, and early enteral nutrition have been evaluated for improving short-term outcomes of esophagectomy. ERAS protocols resulted in faster recovery from surgery and reduced morbidity rates in observational studies [17]. RCTs have been done only rarely on aspects of ERAS protocols after esophagectomy [18]. Overall, ERAS protocols can be judged as safe, feasible, and promising, but without an evidence-based effect on patient outcomes [19].

## 11. Conclusions

The presented results show HMIE as a highly reliable technique, not only for the resection part, but especially in terms of safety in reconstruction and anastomosis for minimally invasive esophagetomy. For esophageal surgeons with experience in minimally invasive anti-reflux procedures and obesity surgery, HMIE is easy and fast to learn and can be adopted without significant learning curve associated morbidity.

## Figures and Tables

**Table 1:** Clinicopathological factors.

	All (*n* = 157)
**Male gender (*n*, %)**	76% (119)
**Age (median (range), years)**	62 (30–91)
**BMI (median (range), kg/m^2^)**	26 (18–49)
**ASA-Score (*n*, %)**	
1–2	49% (77)
3–4	51% (80)
**Comorbidity (*n*, %)**	
History of smoking	37% (58)
Pulmonary	31% (48)
Cardiac	20% (32)
**Tumor location (*n*, %)**	
Lower 1/3	85% (134)
Middle 1/3	14% (22)
Upper 1/3	0.6% (1)
**Tumor histology (*n*, %)**	
Adenocarcinoma	76% (119)
Squamous cell carcinoma	20% (32)
Other Tumor	3% (4)
Benign	1.3% (2)
**Neoadjuvant treatment (*n*, %)**	
None	22% (35)
PeriCTX	48% (75)
NeoRCTX	30% (47)
**Postoperative UICC stage (*n*, %)**	
Not applicable (benign)	1.3% (2)
0/1	55% (87)
2	17% (26)
3	20% (32)
4	6% (10)

Scale variables were expressed as median and range, ordinal and nominal parameters as absolute numbers, and percent. BMI: Body mass index; ASA: American Society of Anesthesiologists; UICC: Union international contre le cancer.

**Table 2:** Procedure and hospital stay characteristics.

	All (*n* = 157)
**Lymph nodes harvested (*n*)**	23 (3–58)
**Negative Resection margin (R0)**	94% (147)
**Operating time (min)**	290 (147–507)
**Perioperative blood transfusion required (*n*, %)**	14% (22)
**Conversion required**	1.3 % (2)
**IMC stay (days)**	5 (3–65)
**Hospital stay (days)**	14 (7–91)

Scale variables were expressed as median and range, ordinal and nominal parameters as absolute numbers, and percent. IMC: intermediate care unit.

**Table 3:** Surgical outcome and postoperative complications.

	All (*n*=157)
**Perioperative morbidity**	54% (85)
**Surgical morbidity**	29% (46)
Anastomotic leakage	2% (3)
Gastric Condiut Necrosis	0.6% (1)
Mucosal Ischemia	2.5% (4)
Chylothorax	3% (5)
Wound infection	3% (5)
Delayed gastric emptying	17% (27)
**Pulmonary morbidity**	31% (49)
Pneumonia	17% (27)
Pleural effusion	15% (23)
**Cardiac Complications**	15% (23)
**Severity of complications**	
Grade 0	46% (72)
Grade I; II	20% (32)
Grade IIIa/b	28% (44)
Grade IVa/b; V	6% (9)
**90 days mortality**	2.5% (4)

Scale variables were expressed as median and range, ordinal and nominal parameters as absolute numbers, and percent. Complications were graded according to Clavien/Dindo Classification.

## Abbreviations

TMIE	Total minimally invasive esophagectomy
HMIE	Hybrid minimally invasive esophagectomy
OE	Open esophagectomy
PDS	Polydiaxone suture
RCT	Randomized controlled trial
CT	Computed tomography
ERAS	Enhanced recovery after surgery
